# Antimicrobial activity and chemical composition of white birch (*Betula papyrifera* Marshall) bark extracts

**DOI:** 10.1002/mbo3.944

**Published:** 2019-10-03

**Authors:** Dorian Blondeau, Annabelle St‐Pierre, Nathalie Bourdeau, Julien Bley, André Lajeunesse, Isabel Desgagné‐Penix

**Affiliations:** ^1^ Department of Chemistry, Biochemistry and Physics University of Québec at Trois‐Rivières Trois‐Rivières QC Canada; ^2^ Innofibre Cégep of Trois‐Rivières Trois‐Rivières QC Canada; ^3^ Groupe de Recherche en Biologie Végétale University of Québec at Trois‐Rivières Trois‐Rivières QC Canada

**Keywords:** antimicrobial activity, bark extracts, *Betula papyrifera* Marshall, mass spectrometry, white birch

## Abstract

Extracts from white birch have been reported to possess antimicrobial properties, but no study has linked the chemical composition of bark extract with antimicrobial activity. This study aimed to identify white birch (*Betula papyrifera* Marshall) bark extracts with antimicrobial activity and elucidate its composition. In order to obtain the highest extraction yield, bark residues >3 mm were retained for extraction. A total of 10 extraction solvents were used to determine the extraction yield of each of them. Methanol and ethanol solvents extracted a greater proportion of molecules. When tested on eight microorganism species, the water extract proved to have the best antimicrobial potential followed by the methanol extract. The water extract inhibited all microorganisms at low concentration with minimal inhibitory concentration between 0.83 and 1.67 mg/ml. Using ultraperformance liquid chromatography coupled to a time‐of‐flight quadrupole mass spectrometer, several molecules that have already been studied for their antimicrobial properties were identified in water and methanol extracts. Catechol was identified as one of the dominant components in white birch bark water extract, and its antimicrobial activity has already been demonstrated, suggesting that catechol could be one of the main components contributing to the antimicrobial activity of this extract. Thus, extractives from forestry wastes have potential for new applications to valorize these residues.

## Graphical abstract

We have shown that several extracts of white birch bark residues display natural antimicrobial activity. Among them, the water extract followed by the methanol extract proved to have the best antimicrobial potential. For the first time, extracts were characterized using ultrahigh‐performance liquid chromatography‐quadrupole time‐of‐flight mass spectrometry, which led to the identification of several molecules including catechol, predominant in the water extract, which is known to have antimicrobial activity.

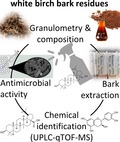

## 1 INTRODUCTION

More than 50 million tons of bark, mainly derived from pulp and wood industries, are produced annually in North America (Gupta, 2009). In Canada, only a fraction of the bark is used as an energy source by direct combustion, and the rest of the bark is incinerated or landfilled as waste (Cheng, Deng, Zhang, Riedl, & Cloutier, 2006). Both incineration and landfilling are nonsustainable avenues, whereas the combustion of bark is not ideal for energy production as it contains a high ash content that lower its heating values. Thus, the combustion of bark for energy recovery is not economically advantageous. In addition to ash, cellulose, hemicellulose, and lignin, bark also contains small amounts of bioactive compounds called extractives, which have potential to provide value‐added coproducts to bark. Recent studies have revealed that forestry wastes such as bark possess potentially important properties for new applications, which are related to their chemical content (Feng, Cheng, Yuan, Leitch, & Xu, 2013; Jablonsky et al., 2017).

The biodiversity of the boreal forest constitutes an important pool of natural bioactive compounds (Royer, Ben Amor, Boucher, & Les, 2016; Royer, Houde, Viano, & Stevanovic, 2012). For example, the white birch (*Betula papyrifera* Marshall), one of the broad‐leaved species widely present in the boreal forest of North America, contains a large amount of bioactive molecules including terpenoids and phenolic compounds known to have antimicrobial and antioxidant properties (Mshvildadze, Legault, Lavoie, Gauthier, & Pichette, 2007; Royer et al., 2012). Bark is a set of dead tissues, developed after the primary and secondary growth of bark, which together form the protective layers of branches and the trunks of woody plants. The bark inhibits water loss through evaporation and has a protective role against overheating, frost, herbivores, or pathogens. It comprises up to 20% of the dry weight of woody plants and contains various molecules (Tanase, Coșarcă, & Muntean, 2019). The extraction of such molecules for the valorization of bark residues prior to thermal energy production is an interesting way to make it economically and environmentally advantageous for several industries (e.g., pharmaceutical, cosmetic, food, and sanitary industries). This important and inexpensive source of bioactive molecules can be used in the formulation of biosourced products, thus promoting applications for forest industries. As a result of the industrial method of wood processing, bark residues are removed from the sapwood and discarded (Celhay, Mathieu, Candy, Vilarem, & Rigal, 2014). For these reasons, residues are present in large quantities, thus representing an abundant and currently underutilized natural resource (Zhao, Yan, & Cao, 2007). Currently in Québec, the only valorization of bark is its unefficient transformation into heat and electricity by cogeneration central. It is therefore necessary to find new ways to valorize this biomass, including the production of biosourced products.

The use and research for new drugs, food supplements, and sanitary products derived from plants has continuously increased in recent years (Jamshidi‐Kia, Lorigooini, & Amini‐Khoei, 2018). In particular, natural products represent new avenues for the treatment of infectious diseases (Saleem et al., 2010). While 25%–50% of current pharmaceuticals come from plants, few are used as antimicrobial agents (Gurib‐Fakim, 2006). Plants are known to be rich in a wide variety of specialized (aka secondary) metabolites (e.g., phenolics, terpenoids, and alkaloids), often studied in vitro for their antimicrobial properties (Cowan, 1999; Royer, Prado, García‐Pérez, Diouf, & Stevanovic, 2013). These specialized metabolites are produced in various plant tissues whose main function is to protect the plant against fungi, bacteria, and insect attacks (Omar et al., 2000; Wink, 1988). For example, the antimicrobial activity of plant phenolic compounds such as catechin and ellagitannins has been intensively studied (Chandra et al., 2017; Daglia, 2012; Puupponen‐Pimiä, Nohynek, Alakomi, & Oksman‐Caldentey, 2005; Zhang et al., 2016). In addition to controlling invasion and growth of plant pathogens, terpenoids (thymol and carvacrol) were found to be active against human pathogens as well (Barbieri et al., 2017; Moon & Rhee, 2016). The use of plant specialized metabolites as antimicrobial compounds tends to increase due to the constant emergence of microorganisms resistant to current antimicrobial agents (Amábile‐Cuevas, 2003; Saleem et al., 2010). However, the majority of these studies focused on flowering plants and only few studied woody plants or forest residues (Annabelle, Dorian, Nathalie, Julien, & Isabel, 2019; Papuc, Goran, Predescu, Nicorescu, & Stefan, 2017; St‐Pierre et al., 2018; Tanase et al., 2018). It is therefore essential to investigate the antimicrobial potential of specialized metabolites present in bark residues of Québec's forest industries to valorize these residues and to expend the repertoire of antimicrobial compounds.

White birch is an important source of extractives with many interesting biological properties for the formulation of high value‐added coproduct (Krasutsky, 2006). Birch bark is a low‐value waste product in the forest industry (Ekman, 1983). In fact, 96,000 tons of paper birch bark is produced annually in Québec province (Pedieu, Riedl, & Pichette, 2009). The majority of extractives from this species is obtained from its bark with a yield of 22 g/100 g of dry bark (Krasutsky, 2006). Previous studies on white birch showed that specialized metabolites (e.g., terpenoids and polyphenols) from this tree possess several beneficial pharmacological properties (Gauthier, Legault, Lebrun, Dufour, & Pichette, 2006; Krasutsky, 2006; Vandal, Abou‐Zaid, Ferroni, & Leduc, 2015). For example, pentacyclic triterpenoids, mainly of the lupane and oleanane types, have been isolated from the outer bark of various species of birch, including *Betula papyrifera* Marshall. These triterpenoids displayed diversified biological activities such as bactericidal, antiviral, anti‐inflammatory, cytotoxic, and anticancer (Gauthier et al., 2006; O'Connell, Bentley, Campbell, & Cole, 1988; Omar et al., 2000). Specifically, betulin found in large quantities in the bark of white birch (72.4%) and betulinic acid (5.4%) are two lupane triterpenoids with great interest for the pharmaceutical sector because of their antimicrobial, anticancer, and anti‐HIV properties (Krasutsky, 2006; Royer et al., 2016). In addition, birch phenolic compound platyphylloside was shown to exert anticancer activity in vitro against lung carcinoma (A‐549) and colorectal adenocarcinoma (DLD‐1) human cell lines (Mshvildadze et al., 2007). Although the antimicrobial potential of a few specialized metabolites from white birch has been reported, the composition of bark extractives and its correlation with antimicrobial activity has not yet been investigated.

The objective of this research was to extract the specialized metabolites present in the bark of white birch and to determine the optimal conditions to maximize extraction yields either by using different solvents or different bark granulometries. Initial screening of specialized metabolites was performed using thin‐layer chromatography coupled with different colorimetric revelation methods. In addition, the antimicrobial activity of the extracts was measured against eight different microorganisms using the broth microdilution method. The minimal inhibitory concentration (MIC) as well as the minimal bactericidal/fungicidal concentration (MBC/MFC) was evaluated. Finally, the chemical composition of birch extracts with antimicrobial potential was determined using ultraperformance liquid chromatography coupled to a time‐of‐flight quadrupole mass spectrometry (UPLC‐QTOF‐MS). The characterization of the compounds present in each extract allows to identify molecules with an associated antimicrobial activity. Consequently, biologically active molecules obtained from the bark of white birch could be exploited on an industrial scale in order to valorize these abundant residues from forest wastes.

## 2 MATERIALS AND METHODS

### 2.1 Material and reagent

A total of 10 solvents were used for the extraction of bark residues. The majority were purchased from Fisher Scientific (methanol HPLC Grade, ethanol denatured, acetone certified ACS, hexane HPLC Grade), while the others (chloroform 99.9%, methylene chloride >99%, ethyl acetate 99.6%) were obtained from Acros Organics. For the acid–base extraction, hydrochloric acid (Fisher certified ACS) was used to acidify methanol at pH 4, as well as 7 N ammonia (Acros organics) to alkalize the aqueous fraction during liquid–liquid extraction. For the revelation of thin‐layer chromatography (TLC) plates, reagents were purchased from Fisher Chemical. All extracts were dissolved in dimethyl sulfoxide (DMSO), certified ACS from Fisher Chemical.

### 2.2 Plant material and granulometric fraction

White birch (*B. papyrifera* Marshall) bark was collected from the Thomas‐Louis Tremblay industry, a sawmill from Ste‐Monique in Lac‐St‐Jean (Québec, Canada), during the winter of 2016. The collected bark residues were immediately dried at room temperature. Before starting the laboratory tests, we determined the contribution of bark residues particle size to the total extractives. To do this, bark residues, which also contained wood particles, were sieved with different sizes of strand (3 mm, 7 mm, 45 mm). Fraction sizes were classified in four groups: smaller than 3 mm (<3 mm), between 3 and 7 mm (3–7 mm), between 7 and 45 mm (7–45 mm) and over 45 mm (>45 mm). The largest bark residues in this last group were approximately 300 mm. All fractions were extracted with either of the three solvents: water, water–ethanol, and ethanol. The results are reported as g of extractives in a given fraction group per gram of total mass of the bark residues before sieving. This is to reflect the weight of the fraction in addition to the extractives yield of the fraction (ex. g of 3–7mm extractives/total mass of bark residues batch obtained from the sawmill).

### 2.3 Bark composition

The general composition of the bark residues, which comprised wood particles, ash, extractives, lignin, cellulose, and hemicellulose content, was determined using the National Renewable Energy Laboratory (NREL) protocols: TP‐510‐42618, TP‐510‐42619, TP‐510‐42620, and TP‐510‐42622 (Hames et al., 2008; Sluiter, Hames, et al., 2005; Sluiter et al., 2008b; Sluiter, Ruiz, Scarlata, Sluiter, & Templeton, 2005). Briefly, the method for determining the ash composition was based on the percentage of residue remaining after dry oxidation at 550–600°C. All results are reported relative to the 105°C oven dry weight of the sample. The extractive composition was carried out using a Soxhlet apparatus with ethanol at reflux for 16–24 hr. The lignin content was measured by UV‐Vis spectroscopy (Hach DR6000) after a two‐step acid hydrolysis to fractionate the biomass into forms that are more easily quantified. The monomeric sugars constituting cellulose and hemicellulose were quantified by ion chromatography (Dionex ICS‐5000) with the Dionex CarboPac SA‐10 column and the electrochemical detector.

### 2.4 Bark extraction

The bark was grounded into a fine powder (0.425 mm) by using a Wiley mill. Ten different solvents were used with three solvent‐dependent extraction techniques: water (1), methanol (2), ethanol (3), acetone (4), methylene chloride (5), ethyl acetate (6), chloroform (7), hexane (8), water–ethanol (9), and acid–base (10). The last solvent corresponds to a liquid–liquid extraction technique using several solvents described by Yubin, Miao, Bing, and Yao (Yubin, Miao, Bing, & Yao, 2014). This method allows mainly the extraction of alkaloids from the bark. The majority of the extracts (2, 3, 4, 5, 6, 7, 8) were obtained using the soxhlet extraction technique during 7 hr of reflux. The other two extracts (1, 9) were obtained using an accelerated solvent extractor (Dionex™ ASE™ 350; ThermoFisher). The solid bark was extracted with distilled water at 100°C and 1,500 psi over six cycles of 10 min each. For the water–ethanol extract (9), a liquid extraction with ethanol was carried out following a first extraction with water. The liquid extract was re‐extracted with ethanol at 120°C and 1,500 psi over six cycles of 5 min. Finally, all extracts were evaporated to dryness in a low temperature oven.

### 2.5 Thin‐layer chromatography method

The 10 extracts were dissolved in their respective solvents to a final concentration of 10 mg/ml, and these solutions were used for preparative thin‐layer chromatography. For that purpose, TLC (Aluminum TLC Silica gel 60 F_254_) plates of size 14 × 20 cm were used. Drops (7 μl) of each extract solution were loaded individually onto the baseline of the layer, which was then developed with chloroform: methanol (9:1 v/v). A solution containing several standards concentrated at 1,000 ppm (piperine, vanillin, ferulic acid, glucose, and betulin) was prepared. On each TLC, 7 μl of this solution was deposited in order to visualize the separation of different families of compounds. TLC was dried, and observed in a UV chamber at 254 nm, which revealed the presence of several chemical revelators. The revelators used were a ρ‐anisaldehyde sulfuric acid solution to visualize all types of compounds present in the extract (Jork, Funk, Fischer, Wimmer, & Burns, 1990), iron chloride reagent (FeCl_3_) to observe the presence of phenols (Jork et al., 1990), and Dragendorff reagent for alkaloid compounds (Sasidharan, Chen, Saravanan, Sundram, & Latha, 2011).

### 2.6 Microorganism cultures

The white birch extracts were individually tested against a panel of microorganisms including Gram‐negative strains *Escherichia coli* ATCC 35218, *Salmonella enterica* ATCC 10708, and *Pseudomonas aeruginosa* ATCC 1542; Gram‐positive strains *Staphylococcus aureus* ATCC 6538 and *Enterococcus faecalis *ATCC 29212; fungal strains *Aspergillus niger* ATCC 10535; and yeast strains *Candida albicans* and *Saccharomyces cerevisiae.* The two yeast strains were supplied by the microbiology laboratory of the Université du Québec in Trois‐Rivières (Québec, Canada). Bacterial strains were cultured overnight at 37°C in Mueller Hinton agar (MHA) prior to the antimicrobial tests. Fungus and yeast (fungi) strains were cultured for 72 hr at 37°C in Sabouraud dextrose agar (SDA).

### 2.7 Antimicrobial assays

The antimicrobial activity of the extracts was studied using the broth microdilution method, with appropriate culture media. For example, Mueller Hinton broth (MHB) was used for bacteria and Sabouraud dextrose broth (SDB) for fungi. Microorganism suspensions were prepared in sterile 0.9% saline solution to obtain a final inoculum estimated at 1.5 × 10^8^ cfu/ml for bacteria, 1.5 × 10^6^ cfu/ml for yeast, and 1.5 × 10^4^ cfu/ml for fungus, according to 0.5 McFarland turbidity value as measured using turbidimeter (Hach, 2100AN). Extracts were prepared and dissolved in DMSO at a concentration of 10 mg/ml. According to the antimicrobial testing method, the highest DMSO concentration found in the microplate well was 26%, which had no influence on microbial growth of the tested microorganisms.

To test the antimicrobial properties of the extracts, 96‐well plates were prepared according to a modified method mentioned in Balouiri, Sadiki, and Ibnsouda (2016). Initially, screening of the extracts using 4.5% was carried out in order to quickly identify the potential extracts that allow inhibition of the microbial growth. These extracts were further tested using microdilution technique. The microdilution method was initiated by dispensing 100 μl of each extract in the first column of a 96‐well plate containing 50 μl of broth (MHB or SDB). Serial dilutions of the extracts were carried out in order to obtain a final concentration between 4.44 and 0.01 mg/ml. An equal volume (50 μl) of microbial suspension was added into the wells. Negative and positive controls were prepared without antimicrobial agent and with quaternary ammonium solution (BTC 2125M‐80%) obtained from Sani Marc Group, respectively. The plates were incubated at 37°C during 3 hr for bacteria and 6 hr for fungi. Then, to indicate the metabolic activity of the microorganisms, 40 μl/well of INT (2‐*p*‐iodophenyl‐3‐*p*‐nitrophenyl‐5‐phenyl tetrazolium chloride; Sigma) dissolved in water (2.85 mg/ml) was added to each well. This tetrazolium salt is reduced to red formazan dye by the active dehydrogenases of living cells. The visual development of color was observed after incubation under appropriate cultivation conditions during 1 hr for bacteria and 16 hr for fungi. MIC values were defined as the lowest concentration of each natural product which resulted in no color formation, meaning that microbial growth was inhibited. Results were expressed in milligrams per milliliters. All measurements of MIC values were repeated in duplicate. To determine minimum bactericidal/fungicidal concentration (MBC/MFC), an aliquot (100 μl) of each incubated well, with concentration equal or over the MIC, was plated onto MHA for bacteria and SDA for fungi. MBC and MFC were defined as the lowest concentrations that allow no visible growth on agar after 24 hr of incubation for bacteria and 48 hr for fungi.

### 2.8 UPLC‐QTOF‐MS analysis

These analyses were carried out externally by the Centre de Recherche Industrielle du Québec (CRIQ). Briefly, a UPLC analysis was performed using a Waters Acquity Ultraperformance LC system (Waters), equipped with a binary pump system (Waters). An Acquity Ethylene Bridged Hybrid (BEH) C18 column (100 mm_2.mm id, 1.7 mm particle size) from Waters was used. The molecules were separated with a mobile phase that consisted of 0.2% acetic acid (eluent A) and acetonitrile (eluent B). The flow rate was 0.2 ml/min and the gradient elution was initial, 2% B; 0–1 min, 2%–100% B; 1–30 min, isocratic 100% B; 30–33 min, 100%–2% B; 33–33.5 min, isocratic 2% B; 33–40 min. The mass spectrometry (MS) analyses were carried out on a QTOF Micro mass spectrometer (Waters) equipped with a Z‐spray electrospray interface. Each analysis was performed in both positive and negative mode, and the data were acquired through a mass scan from 100 to 1,250 m/z. The ionization occurred at 120°C using a cone gas flow rate of 50 L/hr, desolvation gas flow rate of 350 L/hr, and a desolvation temperature set at 200°C. Nitrogen (99% purity) was used as a nebulizing gas. Data interpretation was carried out with the MassLynx 4.1 software. Mass extraction, deconvolution, and isotope and library search were performed using MZMine 2 (Pluskal, Castillo, Villar‐Briones, & Orešič, 2010).

### 2.9 Statistical analysis

Extraction experiments were carried out in triplicate. Data were expressed as mean ± standard deviation. Significant differences (*p* < .05) among the mean values of extraction results were determined by one‐way analysis of variance (ANOVA) followed by Tukey test, using the Past 3 statistical software (version 3.16; Hammer, Harper, & Ryan, 2001).

## 3 RESULTS

### 3.1 Granulometry, composition, and extraction yields

**Figure 1:**
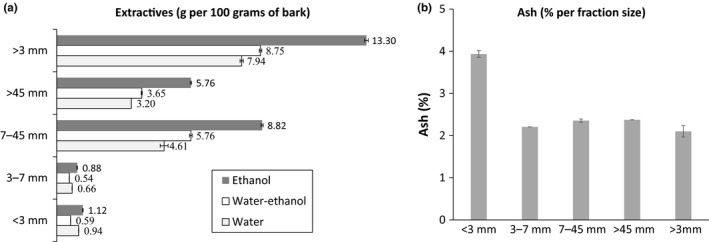
Analysis of bark biomass. Table of the optimal granulometric fraction to be used for the extraction of the bark (a), the graph shows the extractive concentration (g) per 100 g of bark batch among fraction sizes following extraction with ethanol, water–ethanol, and water. The graph (b) represents the percentage of ash in the bark fraction <3 and 3–7 mm

The residues of white birch bark were sieved using different sieve sizes to obtain four fractions of bark residue particle sizes. The different fractions were crushed and extracted using three solvents: water, water–ethanol, and ethanol. The extraction yields (g/100 g of total bark residues) obtained for each granulometric fraction of bark are summarized in Figure 1a. The fraction >3 mm represents the mix of three fraction groups: 3–7, 7–45, and >45 mm. In general, extraction with ethanol yielded a greater extractive content compared to other solvents (Figure 1a). With regard to the extraction yield by function of the particle size distribution, smaller fractions (3–7 and <3 mm) contributed to a smaller amount of the extractive yield compared to the larger ones (7–45 and >45 mm). For example, the fraction 7–45 and >45 mm extracted with ethanol yielded, respectively, 8.82 and 5.76 g of extractives/100 g of bark, whereas the fractions <3 and 3–7 mm generated 8 times less with only 1.12 and 0.88 g/100 g of bark, respectively (Figure 1a).

Next, we quantified the ash content present in each particle size fraction of bark residues, and the values obtained are presented in Figure 1b. The ash content of fraction <3 mm (3.94%) was significantly higher compared to others, which were 2.21% for 3–7 mm, 2.36% for 7–45 mm, and 2.37% for >45 mm.

**Figure 2:**
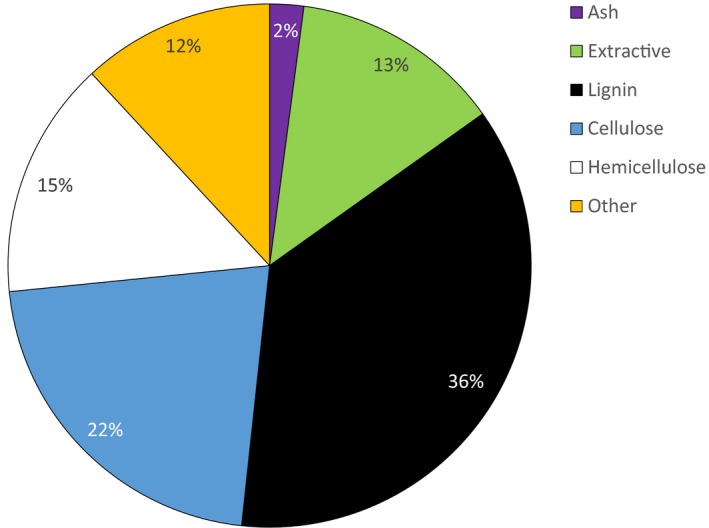
Pie chart of bark components. The pie chart represents the analysis of the composition of the bark in fraction >3 mm

Next, the general composition of white birch bark residues was assessed. The values are reported as a percentage of the total mass of the whole fraction >3 mm. The largest proportion of the composition corresponded to lignin (36.55%), followed by cellulose (21.70%) and hemicellulose (14.70%). In addition, the average extractive content following ethanol extraction was 13.05% (Figure 2).

**Figure 3:**
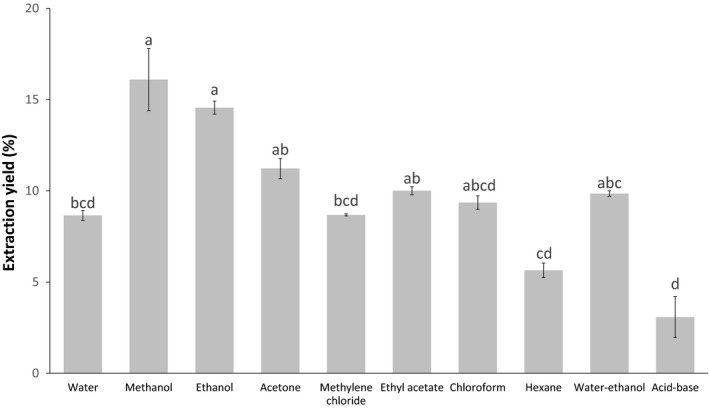
Bark extraction yields. Graph of the extraction yield according to the solvent used

The extraction yields of the residues as a function of the solvent used are reported in Figure 3. The results show that alcohol solvents were more effective and extracted more compounds altogether, with the methanol and ethanol extracts having a respective percent extraction of 16.10% and 14.56% (Figure 3). These two polar solvents extracted more compounds present in residues, whereas the less polar solvents (e.g., hexane 5.64% yield) had lower extraction yields. In addition, the acid–base extraction protocol aimed to extract alkaloid compounds displayed the lowest extraction efficiency with 3.09% and appeared to be the least optimal condition to obtain the highest weight of dry extractive (Figure 3).

### 3.2 Metabolite profile—Thin‐layer chromatography

**Figure 4:**
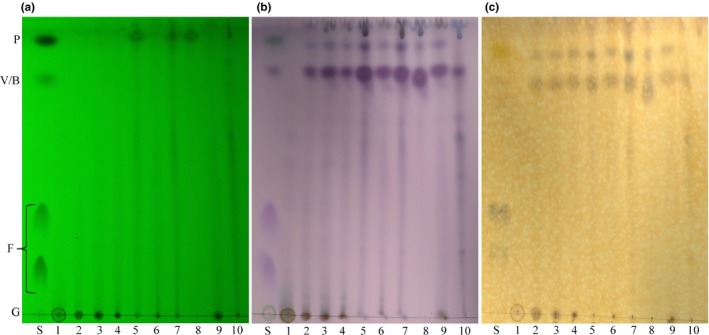
Thin‐layer chromatography of bark extracts. Thin‐layer chromatography of bark extracts with chloroform: methanol (9:1) and visualized under UV 254 nm (a), stained with ρ‐anisaldehyde (b) and with FeCl_3_ (c). Lane S corresponds to a mixture of standards (P, piperine; V, vanillin; B, botulin; F, ferulic acid; and G, glucose). The samples from left to right were extracts from different solvents: water (1), methanol (2), ethanol (3), acetone (4), methylene chloride (5), ethyl acetate (6), chloroform (7), hexane (8), ethanol–water (9), and acid–base (10)

Next, we assessed the general chemical composition of crude white birch bark extracts using thin‐layer chromatography (TLC) (Figure 4). The TLC plates correspond to the chromatogram showing the separation of compounds present in each extract using different revelation reagents. Based on the mobile phase used, most of the polar compounds present in the extracts were found at the bottom of the TLC plate, while less polar compounds migrated to the top of the silica TLC plate. With the help of standards, this technique made it possible to roughly visualize which types of molecules were present in each extract according to their polarity. Each TLC plate showed a lane of each extract (1–10) and a lane containing four standards (S) (P: piperine, V: vanillin, B: betulin, F: ferulic acid, G: glucose) (Figure 4). The first TLC plate showed compounds that reacted with UV (λ = 254 nm), in other words, aromatic compounds having a strong conjugation in their molecular structure (Figure 4a). On this plate, only three standards were visualized, piperine (P), vanillin (V), and ferulic acid (F), which can be attributed to their chemical structure. On the second TLC plate, separated compounds reacted with the ρ‐anisaldehyde reagent to demonstrate the presence of different families of compounds (Figure 4b). Each of these families reacted differently and can be distinguished by their different colors. The colored compounds at the bottom of the plate are strongly polar. For example, sugars such as glucose (G) show a green spot in the standard column (S), while the compounds higher on the plate are less polar, such as betulin (B), a triterpenoid characterized by a purple spot. Results showed that extracts using polar solvents such as water, methanol, and ethanol contained a greater proportion of sugars (green‐brown spots) than the other extracts. Moreover, all extracts appeared to have several nonpolar compounds including terpenoids (purple spot) and phenolic compounds. The third TLC plate showed the phenolic compounds which reacted with iron chloride (FeCl_3_; Figure 4c). Phenolic compounds in the extracts had more affinity for the mobile phase and were concentrated at the top of the TLC plate. By comparing the last two TLC plates, it is interesting to note that the two lines of purple spots at the top of the second plate likely corresponded to phenolic compounds, since they are found at the same position (*R_f_* of 0.8 and 0.9; Figure 2b) than the spots at the top of the third plate that reacted with FeCl_3_ (Figure 4c). In addition, the Dragendorff reagent, used to visualize the presence of alkaloids, was applied to a TLC plate. However, the results showed a very low presence (accumulation and number) of these compounds in the white birch extracts.

### 3.3 Antimicrobial activity

Using the broth microdilution method, the antimicrobial activity of the ten extracts was determined on eight microorganisms. Table 1 shows the results obtained in regard to the antimicrobial power of bark extracts on five bacteria and three fungal species according to the extraction solvent used. A quaternary ammonium compound (QAC, BTC 2125M‐80%, Stepan^®^) was used as a positive control because it is a well‐known antimicrobial agent often used as an active ingredient in the formulation of several biocidal products such as surface disinfectants. In addition, for each test, a negative control was included, which contained no agent or extract and resulted in the growth of each microorganism.

**Table 1:** Antimicrobial activity of white birch bark extracts against different strains of microorganisms

Extracts	*Escherichia coli*		*Salmonella enterica*		*Pseudomonas aeruginosa*		*Staphylococcus aureus*	
	MIC^a^	MBC/MFC^b^	MIC	MBC/MFC	MIC	MBC/MFC	MIC	MBC/MFC
Water	1.67	−c	0.83	−	0.83	−	1.67	1.67
Methanol	−	−	1.67	4.44	−	−	4.44	4.44
Ethanol	−	−	1.67	−	1.67	−	−	−
Acetone	−	−	4.44	−	−	−	−	−
Methylene chloride	0.83	−	1.67	4.44	0.21	0.21	−	−
Ethyl acetate	−	−	1.67	−	−	−	−	−
Chloroform	−	−	1.67	4.44	0.21	0.83	−	−
Hexane	−	−	1.67	−	−	−	−	−
Water–ethanol	1.67	−	1.67	−	1.67	−	4.44	−
Acid–base	4.44	−	1.67	−	−	−	−	−
QAC^d^	2.60	5.21	0.65	0.65	10.42	10.42	5.21	5.21

Extracts	*Enterococcus faecalis*		*Aspergillus niger*		*Candida albicans*		*Saccharomyces cerevisiae*	
	MIC	MBC/MFC	MIC	MBC/MFC	MIC	MBC/MFC	MIC	MBC/MFC
Water	1.67	−	0.83	−	1.67	−	1.11	4.44
Methanol	1.67	4.44	1.67	−	−	−	2.22	−
Ethanol	−	−	1.67	−	−	−	2.22	−
Acetone	−	−	1.67	−	−	−	2.22	−
Methylene chloride	−	−	−	−	−	−	1.11	4.44
Ethyl acetate	−	−	−	−	−	−	2.22	−
Chloroform	−	−	−	−	−	−	1.11	4.44
Hexane	−	−	−	−	−	−	−	−
Water–ethanol	−	−	−	−	−	−	−	−
Acid–base	−	−	2.22	−	1.67	−	2.22	4.44
QAC^d^	2.60	2.60	10.42	10.42	5.21	5.21	2.60	2.60

a MIC, minimum inhibitory concentration. Values are given as mg/ml.

b MBC/MFC, minimum bactericidal concentration/minimum fungicidal concentration. Values are given as mg/ml.

c Not active at maximum concentration (4.44 mg/ml)

d QAC, quaternary ammonium cation is used as a positive control (BTC^®^ 2125M‐80%). Values are given as mg/l.

All extracts of white birch bark residues inhibited the growth of *S. enterica*, a Gram‐negative bacterium (Table 1). For this bacteria, the MIC of each extract was 1.67 mg/ml, except for the water and acetone extracts which had a MIC value of 0.83 and 4.44 mg/ml, respectively. In addition, only the extracts with methanol, methylene chloride, and chloroform displayed bactericidal effect on *S. enterica* at a concentration of 4.44 mg/ml. For *E. faecalis* and *C. albicans*, only the water and methanol extracts promoted inhibition, and none caused total kill. The acid–base extract inhibited the growth of five out of eight microorganisms tested and was fungicidal for *C. albicans* with a MFC value of 4.44 mg/ml (Table 1).

Looking at the full range of antimicrobial test results, few extracts stood out and displayed greater potential as an antimicrobial agent. For example, the water extract inhibited all eight microorganisms tested and showed bactericidal effect on bacteria *S. aureus* (MBC 1.67 mg/ml) as well as on yeast *S. cerevisiae* (MFC 4.44 mg/ml). It was therefore the most promising extract because of its broad spectrum of antimicrobial activity at low concentrations, since its MIC was found to be between 0.83 and 1.67 mg/ml. The methanol extract was another promising one, because it inhibited the growth of five microorganisms (*S. enterica*, *S. aureus*, *E. faecalis*, *A. niger,* and *S. cerevisiae*) at MIC of 1.67 mg/ml except for *S. aureus* and *S. cerevisiae* that have MIC values of 4.44 and 2.22 mg/ml, respectively. In addition, this extract displayed bactericidal effect on *S. enterica*, *S. aureus,* and *E. faecalis* at a concentration of 4.44 mg/ml. The extract obtained using the acid–base extraction method, which is specific to enrich alkaloids, also shows a strong antimicrobial activity. It inhibits the growth of five microorganisms (*E. coli*, *S. enterica*, *A. niger*, *C. albicans,* and *S. cerevisiae*) and kills only one group of microorganisms, *S. cerevisiae*, at a concentration of 4.44 mg/ml.

### 3.4 Characterization using UPLC‐QTOF‐MS

After selection of the extracts with the best antimicrobial activities, it was important to identify their exact chemical composition to help target molecules that may be responsible for the biological activity.

The chemical composition of the white birch bark water and methanol extracts analyzed by UPLC‐QTOF‐MS is presented in Tables 2 and 3, respectively. These tables show the different molecules identified using database based on their exact mass. The compounds in the tables were selected on the basis of their relevance and percentage of area under the peak (% area) ≥0.50. The identified compounds were classified into families of molecules. Using this method of analysis, it was possible to notice that the retention time was not similar or even close together between the same family of compounds. This value derives from the affinity of each constituent for the stationary phase, which depends on its solubility in this phase and its polarity. Two modes of ionization were used, namely, the positive mode (M+H) and the negative mode (M−H) to ensure that the majority of compounds were detected and identified during the analysis.

**Table 2:** Composition of white birch bark water extract using UPLC‐QTOF‐MS analyses

Compounds^a^	*R_t_* b	Exact mass (m/z)^c^		Area^d^ (%)
		[M+H]	[M−H]	
Phenols				
Epirosmanol	5.08	—	345.1393	5.88
Sakuranetin	5.64	—	285.1237	1.13
Catechol	5.65	—	109.0359	6.47
4‐Hydroxybenzaldehyde	6.84	—	121.0385	1.65
Fisetin	9.52	287.1035	—	3.75
Scutellarein	12.41	—	285.0584	2.48
Oleuropein‐aglycone	14.61	—	377.1308	0.51
	16.12	—		0.67
Kaempferol	15.55	—	285.0992	3.70
	15.57	287.1035	—	2.86
Piceatannol	16.24	—	243.0846	0.53
Terpenoids				
Terpendole C	7.87	520.3510	—	2.35
Artabsin	8.40	249.1232	—	4.43
Confertiflorin	11.10	307.1928	—	0.88
*p*‐Menthane‐3,8‐diol	11.63	—	171.1150	1.02
Phytuberin	14.30	295.1483	—	1.03
Alkaloids				
Gentianaine	0.98	142.0390	—	2.25
Berberine	4.52	—	335.1533	2.27
Tubulosine	7.53	476.3204	—	2.03
Avenanthramide 2f	13.55	—	329.2584	0.88
Macarpine	14.29	—	391.1157	0.56
(–)‐Solenopsin A	23.78	254.2584	—	4.48
Glycosylated molecules				
3‐Hydroxyphloretin 2'‐*O*‐glucoside	7.16	—	451.1916	0.57
Phlorin	9.51	—	287.0781	1.59
Grandidentatin	9.80	—	423.1843	3.09
	10.15			2.51
Eriodictyol 7‐*O*‐glucoside	10.78	—	449.1708	3.28
	11.93			3.16
Arbutin	12.20	—	271.0804	3.16
	12.22	273.0878	—	3.59
Acids				
Muconic acid	1.12	—	141.0299	0.69
Caffeic acid	1.19	—	179.0726	0.96
Hydroxycaffeic acid	1.21	—	195.0641	0.59
3‐*p*‐Coumaroylquinic acid	5.00	—	337.1743	2.05
5‐*O*‐Galloylquinic acid	5.85	—	343.1214	1.03
Feruloyl tartaric acid	6.23	—	325.1221	0.55
	6.54		325.1133	0.77
Vanillic acid	6.36	—	167.0478	0.50
*p*‐Coumaric acid	7.59	—	163.0480	3.05
4‐Hydroxybenzoic acid	9.14	—	137.0339	3.85
Pisiferic acid	9.21	317.1948	—	0.73
Ellagic acid	13.14	—	301.0882	0.61
Sugars				
l‐Rhamnose	0.98	165.0511	—	0.20
(+)‐Catechin 3‐*O*‐gallate	7.75	—	441.1687	0.72
d‐Glucose	34.86	181.0349	—	0.21
Others				
4‐Nitrophenol	1.02	140.0050	—	0.80
1,4‐Naphthoquinone	3.02	—	157.0604	1.20
Marchantin A	8.92	—	439.1857	1.03
Picrasin C	9.33	—	421.1777	2.61
	34.89		421.2571	0.50
Myristamide	23.05	228.2430	—	3.02
Heptadecylamine	26.02	256.2760	—	3.87
2‐Nitrophenol	34.87	139.9936	—	1.36

a The compounds were selected on the basis of their relevance and having a % area ≥0.50. The compounds were determined by comparing the exact masses in a data bank. Potential identities are presented.

b Retention time (min).

c Exact mass depending on the ionization mode of the analysis, either positive [M+H] or negative [M−H].

d The percentage of area is relative to the ionization mode of the analysis used.

**Table 3:** Chemical composition of white birch bark methanol extract using UPLC‐QTOF‐MS analyses

Compounds^a^	*R_t_* b	Exact mass (m/z)^c^		Area^d^ (%)
		[M+H]	[M−H]	
Phenols				
Pentacosyl resorcinol	7.32	—	459.1974	2.78
Hydroxymatairesinol	7.44	—	373.1544	3.66
Anhydro‐secoisolariciresinol	7.99	—	343.1340	1.36
Taxifolin	8.48	—	303.0838	0.82
Ligstroside	9.26	—	523.2272	2.30
	9.40			2.35
Phloridzin	10.83	—	435.1445	1.44
Dimethylquercetin	13.64	—	329.2446	3.24
Kaempferid	14.20	—	299.1744	3.33
Acacetine	15.69	—	285.0872	3.30
Oleuropein‐aglycone	16.26	—	377.1339	0.65
Terpenoids				
Perilloside	11.61	—	313.1478	5.54
Deoxystansioside	14.53	—	295.1493	3.76
Alkaloids				
Annotinine	7.40	276.1790	—	2.35
Methylconiine	10.76	142.1624	—	1.74
Isocorypalmine	11.14	342.2122	—	4.19
Pinidine	11.65	140.1349	—	6.57
Coniine	14.57	128.1771	—	4.95
Glycosylated molecules				
Galloyl glucose	5.47	—	331.1245	1.20
Hydroxyphloretin‐glucoside	9.65	—	451.1421	1.12
Apigenin 6‐glucoside	10.32	—	431.1400	2.18
Apigenin‐diglucoside	11.13	—	593.2709	2.96
Acids				
Ibotenic acid	1.38	159.0468	—	0.61
Caffeic acid	1.55	—	179.0905	0.84
Hydroxybenzoic acid	6.05	—	137.0502	0.57
	9.89	—	137.0389	0.67
Coumaric acid	7.85	—	163.0530	0.78
Valoneic acid dilactone	8.55	—	469.1547	1.15
Chicoric acid	9.16	—	473.1739	1.74
	9.52			2.80
12‐Hydroxydodecanoic acid	9.58	217.1958	—	0.68
	9.87			0.66
Others				
Carbophenothion	11.14	342.9818	—	1.52
Butonate	12.99	326.9941	—	3.73
Asparagusate	13.64	150.9699	—	3.93
Retinal	20.07	285.1934	—	1.41

a The compounds were selected on the basis of their relevance and having a % area ≥0.50. The compounds were determined by comparing the exact masses in a data bank. Potential identities are presented.

b Retention time (min).

c Exact mass depending on the ionization mode of the analysis, either positive [M+H] or negative [M−H].

d The percentage of area is relative to the ionization mode of the analysis used.

The water and methanol extracts contained a large number of phenolic compounds and acids. With regard to the water extract (Table 2), the presence of 9 phenolics and 11 acids was noted, including epirosmanol, which is present in large proportion (5.88%), and catechol (6.47%). The methanol extract (Table 3) contained a total of 10 phenolic compounds and 7 acids. In addition, among the nonvolatile components identified, pinidine (6.57%) was found to be most dominant compound in the methanol extract of white birch bark. Some acids were present in both extracts including caffeic acid, hydrobenzoic acid, and coumaric acid. However, these three acids were present in higher proportion in the extract with water. Finally, as anticipated it is observed that the water extract contained more sugars and glycosylated molecules compared to the methanol extract (Tables 2 and 3).

Despite several articles reporting the presence of high betulin content of white birch and its bark (Krasutsky, 2006; O'Connell et al., 1988), no betulin and betulinic acid were detected in water and methanol extracts. One possible explanation is that betulin (if present) is at lower concentration, and we did not derivatize prior to analysis. However, betulin was detected in samples extracted with less polar solvent, including the acid–base, ethyl acetate, and hexane extracts.

The chemical characterization of white birch bark extracts made it possible to target molecules with reported antimicrobial properties (Table 4). A total of six phenolics, one alkaloid and five acids, were listed in either the water or methanol extract. However, most of these molecules were found to be more abundant in the water extract. Catechol, also known as pyrocatechol or 1,2‐dihydroxybenzene, is a phenolic organic compound present at 6.47% concentration in water bark extract. This molecule is a monomer of flavan‐3‐ol, one of the components of proanthocyanidin polymers (condensed tannins).

**Table 4:** Compounds present in water and methanol extracts of white birch bark with known antimicrobial activity as reported in the literature

Compound class	Compound name	Extraction solvent		References
		Water	Methanol	
Phenols	Catechol	+ (6.47)^a^	−	Jeong et al. (2009), Kocaçalışkan et al. (2006)
	4‐Hydroxybenzaldehyde	+ (1.65)	−	Chang, Chen, and Chang (2001), Friedman, Henika, and Mandrell (2003)
	Fisetin	+ (3.75)	−	da Costa et al. (2014), Gabor and Eperjessy (1966)
	Phloridzin	−	+ (1.44)	Barreca, Bellocco, Laganà, Ginestra, and Bisignano (2014), Zhang et al. (2016)
	Kaempferol	+ (3.70)	−	Cai and Wu (1996), Calderon‐Montano, Burgos‐Morón, Pérez‐Guerrero, and López‐Lázaro (2011), Tatsimo et al. (2012)
	Piceatannol	+ (0.53)	−	Plumed‐Ferrer et al. (2013), Yim et al. (2010)
Alkaloids	Berberine	+ (2.27)	−	IgbaláChoudhary (1995), Stermitz, Kamm, and Tawara (2000), Yu et al. (2005)
Acids	Caffeic acid	+ (0.96)	+ (0.84)	Aziz, Farag, Mousa, and Abo‐Zaid (1998), Jeong‐Yong, Jae‐Hak, Seong, and Keun‐Hyung (1998), Merkl, Hrádková, Filip, and Smidrkal (2010), Özçelik, Kartal, and Orhan (2011), Stojković et al. (2013)
	Hydrobenzoic acid	+ (3.85)	+ (0.67)	Jeong‐Yong et al. (1998), Merkl et al. (2010)
	Vanillic acid	+ (0.50)	−	Aziz et al. (1998), Delaquis, Stanich, and Toivonen (2005), Merkl et al. (2010)
	Coumaric acid	+ (3.05)	+ (0.78)	Aziz et al. (1998), Lou et al. (2012)
	Pisiferic acid	+ (0.73)	−	Fukui, Koshimizu, and Egawa (1978), Kobayashi, Nishino, Fukushima, Shiobara, and Kodama (1988)

Note

−, not present; +, present in the extract.

a % of area in the extract.

## 4 DISCUSSION

### 4.1 Granulometry, composition, and extraction yields

The first step of the study was to analyze the crude bark residues of white birch. Since these are postindustrial residues, the bark samples received were not perfectly homogeneous and the chips were of different sizes. Pieces of wood could be found in the residues considering the bark has been removed from the sapwood directly in sawmill following industrial mechanical processes. Since the presence of wood in the bark residues can affect the extractives yield, it was therefore essential to determine the yield for each fraction size. The bark particle size fractioning is generally used in the treatment of biomass and may be used for selective enrichment of specific components (Miranda, Gominho, Mirra, & Pereira, 2012, 2013; Silva, Guilbert, & Rouau, 2011). The results presented the extractives yield in proportion to the mass fraction of the total mass of the bark residues batch, reflecting the real contribution to the total yield (Figure 1a,b). Although the contribution of fractions <3 and 3–7 mm to the total yield was lower than fractions over 7 mm, the 3–7 mm fraction has been retained for the study because its lower contribution was mainly due to its lower mass in the total mass of bark residues batch, as well as the <3 mm fraction. The <3 mm was, however, removed because of its higher ash content. Ash is an inorganic, undesirable material produced during extraction process and tends to accumulate in smaller fractions during biomass processing due to its fine size and fragility (Bridgeman et al., 2007; Liu & Bi, 2011). In addition, the smaller particles may correspond to particles (e.g., sand) that can damage the process equipment. On the other hand, it has been shown that the extent of mineral accumulation in bark fractions depends on the species (Miranda, Gominho, Mirra, & Pereira, 2012). The ash content of *B. papyrifera* Marshall was reported as 1.8% of the bark biomass by Corder (1976). This value is similar to that obtained in our study (2.10%).

Although the composition of white birch extracts has been studied before, the composition of raw bark has been much less studied. Harkin and Rowe (1971) reported that the total extractive content is 22.4% and lignin 37.8% (Krasutsky, 2006). Our results are consistent with these studies since the extractive composition obtained is 13.05% and the lignin content is 36.55% (Figure 2). The composition of the bark has not been fully characterized, and a certain proportion (11.89%) corresponds to other components. These compounds include suberinic acids, waxes, and degradation products present in the bark such as hydroxymethylfurfural (HMF) and furfural, as well as other components of interest, such as organic acids and sugar alcohols (Sluiter et al., 2008a).

Methanol and ethanol solvents significantly extracted a greater percentage of molecules (Figure 3). These two solvents allow the extraction of polar compounds, which include polyphenols, sugars, and some organic acids corresponding to a significant proportion of the total content of the extracts (Tables 2 and 3) (Royer et al., 2012). Less polar solvents such as chloroform and hexane extracted waxes and nonpolar compounds such as triterpenoids present at 20%–35% in white birch bark (Krasutsky, 2006). The extraction with hexane (5.64%) and the acid–base extraction (3.09%) are the least optimal methods for obtaining the highest dry weight in extractives. On the other hand, despite the fact that the methanol and ethanol extracts have higher extraction yields, the eight other solvents should not be eliminated since there are no significant differences between these two and those with acetone, ethyl acetate, chloroform, and water–ethanol. The choice of the best solvent should be determined by the molecules it can extract, those that will provide an antimicrobial effect, and its potential uses in industry. It is certain that a very low extraction yield would be unfavorable, since it would require a huge amount of bark residues to achieve an effective concentration of extract to act as antimicrobial agent. However, if the quantity of extract that can inhibit the growth of microorganisms is low, it is possible that this extraction will be still profitable.

### 4.2 Antimicrobial activity

Plants contain bioactive molecules in different tissues and one of the functions of these specialized metabolites is to protect plants against microbial invaders (Vardar‐Ünlü, Silici, & Ünlü, 2008). A study about the antimicrobial activity of extracts of eastern North American hardwood trees demonstrates that bark extracts had higher inhibitory effect against both bacterial and fungal strains tested than wood extracts (Omar et al., 2000). The bark is the outer most protective part of the tree, so it has a greater amount of specialized metabolites with antimicrobial properties since bark is the first barrier to defend the tree against biotic and abiotic stress.

Among the 10 bark extracts studied, the water extract had the most extensive antimicrobial activity on the eight microbial strains tested followed by methanol extract (Table 1). The water extract had a bactericidal effect on two microorganisms in addition to inhibiting the growth of all tested strains. This is linked to the greater capacity of the water to concentrate the pool of active molecules in this extract. In fact, specialized metabolites with an antimicrobial activity appeared to have more affinity for polar solvents such as water and methanol than nonpolar solvents. Furthermore, as water is a low‐cost “green” solvent (better for environment), it would therefore be a solvent of choice for extraction in the industry (Capello, Fischer, & Hungerbühler, 2007). In addition, the acid–base extract, specific to alkaloids, also demonstrated a strong antimicrobial activity. However, this extraction protocol uses solvents harmful to the environment including hexane and chloroform, which is why this extract is considered less interesting in an industrial context of green chemistry compared to the extract using water or methanol.

Aforementioned, not much is known about the antimicrobial properties of white birch extracts and even less from the bark (Vandal et al., 2015). However, it should be noted that Omar et al. (2000) have shown that ethanol extracts of white birch bark exhibited antimicrobial properties against four bacterial species (two Gram‐positive and two Gram‐negative), but no antifungal activity was observed. In our study, we show that the ethanol extract inhibits microbial proliferation of *S. enterica* and *P. aeruginosa*, but no effect was observed on *E. coli* and *C. albicans*. In contrast, several studies highlighted this biological activity in the extracts of yellow birch (*Betula alleghaniensis*) also present in North America. For example, the aqueous bark extract of yellow birch showed antifungal activity against *S. cerevisiae* (MIC 0.1 mg/ml) as well as antibacterial activity against *E. coli* (Royer et al., 2013; Webster, Taschereau, Belland, Sand, & Rennie, 2008). Furthermore, various studies reported on the antimicrobial activity of different parts of white birch, other than bark. For example, Vandal et al. (2015) demonstrated the antimicrobial activity of ethanol extracts of white birch foliage, twigs, branches, and phloem against *S. aureus* and *C. albicans*. It was further shown that methanol extract of air‐dried white birch branches had activity against 6 of 11 tested bacteria (four Gram‐positive, two Gram‐negative) and three fungal species (Borchardt et al., 2008; McCutcheon, Ellis, Hancock, & Towers, 1992, 1994).

In most studies, the antimicrobial activity of white birch is related to the high abundance of triterpenoids such as betulin and lupeol extract with a less polar solvent such as hexane (Krasutsky, 2006; Krasutsky, Carlson, Nesterenko, Kolomitsyn, & Edwardson, 2007). However, the presence of phenolic compounds in water and methanol extracts (more polar solvents) could also be attributed to greater antimicrobial activity as demonstrated by our results also supported by Royer et al. (2012). In agreement with our results, studies on other North American trees such as *Populus* species have shown that flavonoid and esters of phenolic acids are generally regarded to be responsible for the antimicrobial activity (Vardar‐Ünlü et al., 2008). Kedzia, Geppert, and Iwaszkiewicz (1990) reported that the mechanism of antimicrobial activity is complex and could be attributed to synergism between flavonoids, hydroxyacids, and sesquiterpenes.

### 4.3 Characterization of extracts

The water and methanol extracts contained a large proportion of phenolic compounds as well as many organic acids such as hydrobenzoic acid and coumaric acid present in both extracts (Tables 2 and 3). Catechol, a phenolic compound synthesized by the shikimate pathway in plants, was widely present in the water extract (Kocaçalışkan, Talan, & Terzi, 2006). Catechol has been isolated by Kuiters and Sarink (1986) from leaf and needle litter of several deciduous (beech, birch, oak, hazelnut, maple, willow, and poplar) and coniferous trees (spruce‐fir, douglas‐fir, and larch), and there is also an indication that it is synthesized abundantly in onions and released by their outer layer cells (Farkas & Kiraaly, 1962). Furthermore, catechol has been shown to have antifungal effects on *Colletotrichum circinans* (Farkas & Kiraaly, 1962) and significantly inhibited the growth of *Clostridium difficile* and moderately inhibited the growth of *E. coli* ( Jeong, Jeon, Lee, & Lee, 2009). The results of several previous studies indicated that catechol and its derivatives act as antioxidants in eukaryotic cells, thereby preventing degenerative diseases such as cancer and heart disease (Berberian et al., 2007). Thus, the present results suggested that catechol could be a major active component in white birch bark extract contributing to its antimicrobial activity.

More sugars were present in the water extract than methanol, and this is consistent with the findings of Sjöström and Alén (2013), which suggested that carbohydrates have more affinity for water than other solvents because of its strong polarity. In an antimicrobial agent context, it is preferable to use an extract with less proportion of sugars. Indeed, it has been shown that sugars increase microbial growth because it serves as food for bacteria and fungi. On the other hand, when looking at the results obtained from the antimicrobial assays with each extract, we found that the water extract had a strong antimicrobial activity on the majority of the microorganisms tested. So, despite the presence of sugars, the specialized metabolites present in the extract must have a sufficiently high antimicrobial power to allow the inhibition and the death of microbial strains.

The following four polyphenols were found across the genus *Betula*: (+)‐catechin, salidroside, (+)‐rhododendrin, and platyphylloside (Royer et al., 2012). The (+)‐catechin 3‐*O*‐gallate molecule was identified in our water extract. Several studies have shown that (+)‐catechin, a flavonoid produced by the plant was found to have strong antimicrobial properties (Bais, Walker, Stermitz, Hufbauer, & Vivanco, 2002). (+)‐Catechin is also a well‐known antioxidant and acts as a free radical scavenger, antifungal and antitumor agent, and insect repellant agent (Fukuhara et al., 2002; Veluri, Weir, Bais, Stermitz, & Vivanco, 2004). Several glycosylated molecules have been identified in the extracts such as arbutin, a glycosylated hydroquinone (Table 2). Glycosylation is a widespread modification of plant specialized metabolites. It is involved in various functions, including the regulation of hormone homeostasis, detoxification of xenobiotics, and biosynthesis and storage of specialized compounds. From a chemical point of view, sugar conjugation results in both increased stability (through the protection of reactive nucleophilic groups) and water solubility (Gachon, Langlois‐Meurinne, & Saindrenan, 2005). Several of these molecules have some interesting biological activity without its sugar molecule. For example, apigenin, a chemical compound of the family of flavones, a subclass of flavonoids, has been studied for its multiple biological activities such as anti‐inflammatory and antimicrobial properties (Akroum, Bendjeddou, Satta, & Lalaoui, 2009; Basile, Giordano, López‐Sáez, & Cobianchi, 1999; Funakoshi‐Tago, Nakamura, Tago, Mashino, & Kasahara, 2011; Kukić et al., 2008). The apigenin‐6‐glucoside and apigenin diglucoside were identified in our methanol extract (Table 3). In few articles, only some glycosylated molecules have also demonstrated antimicrobial activity, for example, apigenin‐7‐glucoside in Kukić et al. (2008) as well as kaempferol‐3‐*O*‐glucoside in Akroum et al. (2009). Therefore, we cannot exclude the possibility that these molecules may play a role in the antimicrobial activity of the extracts, despite their association to a sugar moiety.

The antimicrobial properties of the molecules present either in the water or methanol extracts, as shown in Table 4, have been identified before (see reference in Table 4), but the mechanisms underlying these properties have seldom been studied. For example, berberine present at 2.27% in the water extract is an alkaloid known as an antimicrobial agent due to its quaternary ammonium salt structure. This compound acts as an antimicrobial agent by binding minor groves of DNA and by regulating the gene expression of microorganisms (Yu et al., 2005). In contrast, caffeic acid, listed in a lower proportion in both extracts (0.96% in water extract and 0.84% in methanol extract), has an antimicrobial mechanism that acts on the cell wall and the cytoplasmic membrane of microorganisms (Perumal, Mahmud, & Ismail, 2017). Several mechanisms of action of antimicrobial agents are currently known, but the ones involved in white birch bark extracts have yet to be elucidated. Each of the targeted molecules in white birch bark extracts may therefore have different antimicrobial mechanisms and these may have synergistic effects. It is well‐known that plant extracts are complex mixtures of phytochemical compounds, which act synergistically together to achieve antimicrobial effect (Burt, 2004; Cushnie & Lamb, 2011).

The composition of the extracts determined by UPLC‐QTOF‐MS makes it possible to better understand which molecules contribute to their antimicrobial action. However, it is very important to note that the proportion of each component may vary from one tissue specimen to the other within a single species, and vary also due to other parameters such as harvest time, geographical location, or other environmental conditions (Royer et al., 2012; Vardar‐Ünlü et al., 2008). Furthermore, although several samples were pooled, only one method of analysis was performed to identify the compounds present in the extracts (UPLC‐QTOF‐MS). Hence, it is possible that some volatile compounds have not been identified and may play a role in the antimicrobial activity of the extract. In addition, the percentages of area are relative to the mode of analysis used (positive or negative) as well as the ability of a molecule to ionize easily or not. The compounds were identified by comparing the exact masses (m/z) and retention time (*R_t_*) obtained in a database; these are only potential identities of molecules with respect to the percentage of similarity. The complete lists of all the molecules identified for the water extract and the methanol extract can be found in additional material (Tables A1‐A4 in Appendix).

Until today, the main way to valorize bark residues is to burn them to generate heat and electricity. Therefore, it is important to ensure that the added valorization step, that is, extraction of bioactive compounds from residues, will be more efficient or will not affect energy yield. It is thus primordial that the calorific value of the material before and after the extraction is similar or only slightly modified. For this reason, heating value tests should be carried out on bark residues after extraction. When wood is burned, it is the structural components of the wood that burn and produce heat. The high proportions of lignin, cellulose, and hemicellulose (Figure 2) suggest a high calorific value of the material as well as low ash content. In addition, in the case of birch bark, the extractive compounds (Figure 2) may include suberin and betulin, which are the main components giving 1.5 times higher heating value in comparison with wood components (Ferreira et al., 2013; Heinamaki et al., 2017). Several studies have shown that there was a highly significant linear correlation between the higher heating value of the extractive‐free wood and lignin content (Demirbaş, 2001; White, 1987) and another one showed that high ash content in parts of a plant makes it less desirable as fuel (Demirbas, 2002). Despite the fact that some articles demonstrate a slight decrease in the calorific value of wood after extraction, the energy loss is, however, low compared to the added value of the extracted molecules. The production of high value‐added products from white birch bark extract will promote an innovating industrial sector, generating wealth and sustainable jobs, which will encourage wider opportunities to use wood as a renewable crude material (Royer et al., 2012).

To our knowledge, the present study is first to highlight the potential of white birch bark extracts, obtained from sawmill bark residues, as a natural source of antimicrobial agents which could be considered as good candidates for the development of high value products such as new cosmetics, nutraceuticals, or sanitary products. The water extract had the best antimicrobial potential followed by methanol extract. In an industrial context, the water extract is to be prioritized because of its low environmental impact. Using UPLC‐QTOF‐MS, catechol was identified as one of the main components in white birch bark water extract, and its antimicrobial activity has already been studied, suggesting that catechol could be one of the components contributing to the antimicrobial activity of this extract. However, the extract is a complex mixture of phytochemical compounds, which act certainly synergistically together to achieve antimicrobial effect. These results offer the possibility to valorize the bark residues produced in huge quantities by the Canadian forest industry, using the concept of extractives. In addition, the extractive can be obtained while maintaining the biomass that can subsequently be used to energy production and other applications. Nevertheless, further investigations would be required to determine the cytotoxicity of the extracts, their biological efficacies in vivo*,* and their stability in the context of pharmaceutical, cosmetic, food, or sanitary formulations.

With the current challenges that the industrial world faces regarding the unavoidable environmental impact of manufactured goods, industries are turning to sustainable means to reduce this impact and to minimize the damage to the environment while at the same time reaping the marketing bonus that is the claim of a greener product. The bark of woody plants is often considered a forest waste, but it can be an important source of bioactive molecules with a high potential for capitalization. While many studies have focussed on optimizing extraction methods and the identification of bioactive molecules, our study offers additional information on newly identified antimicrobial molecules in white birch bark residues confirming their importance and their value. Future research directions should be directed toward the description of the mechanism of action of these molecules in living systems. Consequently, biologically active molecules obtained from the bark residues could be exploited on an industrial scale.

## CONFLICT OF INTERESTS

None declared.

## AUTHOR CONTRIBUTIONS

DB and AS‐P performed the investigation, analyzed the data, and wrote the original draft. JB and AL provided resources and input on the experimental design, methodology and data analyses. NB and ID‐P conceived, designed the study, and acquired funding. ID‐P revised and edited the manuscript. All authors read and approved the final manuscript.

## ETHICS STATEMENT

None required.

## Data Availability

The datasets used and analyzed during the current study are included in this published article and available from the corresponding author on reasonable request.

## APPENDIX 1

### Crude chemical composition of white birch bark water and methanol extract using UPLC‐QTOF‐MS in positive and negative ionization mode

**Table A1:** Crude chemical composition of white birch bark water extract using UPLC‐QTOF‐MS analyses in positive mode

Exact mass [M+H]	Retention time (*R_t_*)	Compounds	Area under the curve
282.2869	26.5	9‐Octadecenoate	11,588
214.9984	35.6	2,5‐Dioxo‐2,5‐dihydrofuran‐3,4‐diyl diacetate	4,220
254.2584	23.8	(–)‐Solenopsin A	3,785
249.1232	8.4	Artabsin	3,742
563.5732	26.6	Oleic acid, eicosyl ester	3,659
256.2760	26.0	1‐Heptadecanamine	3,269
297.1591	14.4	4‐Prenylresveratrol	3,203
287.1035	9.5	Fisetin	3,166
165.0511	1.0	l‐Rhamnose	2,980
228.2430	23.0	Myristamide	2,545
287.1035	15.6	Kaempferol	2,413
564.3776	8.2	Tris(1‐hydroxy‐2,2,6,6‐tetramethyl‐4‐piperidinyl) phosphate	2,120
184.0484	1.0	3‐Carboxy‐4‐methoxy‐*N*‐methyl‐2‐pyridone	2,029
520.3510	7.9	Terpendole C	1,984
142.0390	1.0	Gentianaine	1,899
608.4124	8.4	C_35_H_61_NO_7_	1,870
476.3204	7.5	Tubulosine	1,715
214.9984	36.2	1‐Deoxy‐d‐xylulose 5‐phosphate	1,636
107.0503	14.4	d‐Glycerate	1,521
267.1002	10.8	Carbamazepine‐*o*‐quinone	1,516
301.0838	15.9	3‐Methoxyapigenin	1,507
652.4316	8.7	1‐Deoxy‐1‐[dodecanoyl(nonyl)amino]‐4‐*O*‐hexopyranosylhexitol	1,478
503.3298	7.9	4a,7b‐Dihydroxy‐3‐(hydroxymethyl)‐1,1,6,8‐tetramethyl‐5‐oxo‐1,1a,1b,4,4a,5,7a,7b,8,9‐decahydro‐9aH‐cyclopropa[3,4]benzo[1,2‐e]azulen‐9a‐yl decanoate	1,233
273.0878	12.2	Arbutin	1,225
139.9936	34.9	2‐Nitrophenol	1,145
459.3102	7.5	Phthalic acid—(8xi,13xi)‐abietan‐18‐ol (1:1)	1,136
547.3581	8.2	Trioctyl trimellitate	1,128
432.3024	7.2	(2beta,3alpha,5alpha,16alpha)‐3‐Hydroxy‐16‐methyl‐2‐(4‐morpholinyl)pregnane‐11,20‐dione	1,125
154.9842	1.0	3,4‐Dihydroxybenzoate	1,108
268.2797	25.2	9‐Octadecen‐1‐amine	959
415.2808	7.2	1‐Methyl‐3‐oxoandrost‐1‐en‐17‐yl heptanoate	911
202.0671	1.0	2'‐Aminobiphenyl‐2,3‐diol	906
295.1483	14.3	Phytuberin	867
696.4679	8.9	quinetolate	830
161.0354	1.0	2‐Oxoadipate	770
296.2758	21.0	*N*‐Hexadecylacrylamide	768
271.1084	13.6	2'‐*O*‐Methylisoliquiritigenin	755
307.1928	11.1	Confertiflorin	745
343.1249	10.0	Coniferin	686
140.0050	1.0	4‐Nitrophenol	673
181.0349	34.9	d‐Glucose	672
149.0252	22.4	trans‐Cinnamate	630
317.1948	9.2	Pisiferic acid	615
124.0296	1.0	Nitrobenzene	580
546.2267	12.9	alpha‐d‐Galactosyl‐*N*‐acetyllactosamine	571
119.0215	1.0	Succinate	516

**Table A2:** Crude chemical composition of white birch bark water extract using UPLC‐QTOF‐MS analyses in negative mode

Exact mass [M−H]	Retention time (*R_t_*)	Compounds	Area under the curve
109.0359	5.7	Catechol	4,973
345.1393	5.1	Epirosmanol	4,517
137.0339	9.1	4‐Hydroxybenzoic acid	2,960
285.0992	15.6	Kaempferol	2,842
271.0804	12.2	Arbutin	2,762
449.1708	10.8	Eriodictyol 7‐*O*‐glucoside	2,523
449.1708	11.9	Eriodictyol 7‐*O*‐glucoside	2,431
423.1843	9.8	Grandidentatin	2,374
163.0480	7.6	*p*‐Coumaric acid	2,344
571.2244	5.1	C_23_H_40_O_16_	2,174
431.1579	10.3	5‐Tricosenylresorcinol	2,089
421.1777	9.3	Picrasin C	2,009
423.1843	10.1	Grandidentatin	1,927
285.0584	12.4	Scutellarein	1,904
299.0783	15.9	Kaempferide	1,763
335.1533	4.5	Berberine	1,742
337.1743	5.0	3‐*p*‐Coumaroylquinic acid	1,572
121.0385	6.8	4‐Hydroxybenzaldehyde	1,267
191.0339	1.3	Scopoletin	1,256
287.0781	9.5	Phlorin	1,222
121.0385	9.4	3‐Hydroxybenzaldehyde	1,116
465.1720	9.5	Dihydromyricetin 3‐*O*‐rhamnoside	1,064
133.0284	1.3	Malate	996
521.1940	7.5	Isobrucein A	971
507.2056	4.8	Gibberellin 2‐*O*‐beta‐d‐glucoside	927
157.0604	3.0	1,4‐Naphthoquinone	926
285.1237	5.6	Sakuranetin	870
439.1857	8.9	Marchantin A	795
343.1214	5.9	5‐*O*‐Galloylquinic acid	791
171.1150	11.6	*p*‐Menthane‐3,8‐diol	781
179.0726	1.2	Caffeic acid	738
175.1160	8.4	Prenylbenzoquinone	685
329.2584	13.5	Avenanthramide 2f	679
587.2112	12.9	Phthalic acid—1,2‐diphenylguanidine (1:2)	603
325.1133	6.5	Feruloyl tartaric acid	590
373.1419	7.1	5‐Nonadecenylresorcinol	550
441.1687	7.7	(+)‐Catechin 3‐*O*‐gallate	550
141.0299	1.1	muconic acid	528
631.2736	5.1	Methyl 1,7,12‐triacetoxy‐17‐(3‐furyl)‐3,11‐dihydroxy‐4,8‐dimethyl‐14,15‐epoxyandrostane‐4‐carboxylate	518
527.1934	12.9	Tremulacin	517
361.1462	2.7	*p*‐HPEA‐EA	516
377.1308	16.1	Oleuropein‐aglycone	515
433.1378	9.2	5‐Tricosylresorcinol	508
117.0299	2.0	Succinate	489
501.2240	8.9	6''‐*O*‐Malonyldaidzin	486
301.0882	13.1	Ellagic acid	472
195.0641	1.2	Hydroxycaffeic acid	455
451.1916	7.2	3‐Hydroxyphloretin 2'‐*O*‐glucoside	434
391.1157	14.3	Macarpine	431
423.2042	9.1	Didrovaltratum	430
485.1945	7.9	Rutaevin	427
393.1261	13.8	Podolactone B	423
325.1221	6.2	Feruloyl tartaric acid	423
405.1508	9.9	Piceatannol 3‐*O*‐glucoside	416
423.1942	9.4	Nigakilactone H	409
239.0911	1.2	4'‐Hydroxyflavanone	409
447.1648	11.4	6‐Hydroxyluteolin 7‐*O*‐rhamnoside	407
243.0846	16.2	Piceatannol	407
377.1308	14.6	Oleuropein‐aglycone	388
421.2571	34.9	Picrasin C	382
167.0478	6.4	Vanillic acid	381
509.1783	17.0	C_30_H_26_N_2_O_6_	377
187.1118	9.6	cis‐2,3‐Dihydro‐2,3‐dihydroxybiphenyl	352
425.1757	7.9	Abscisic acid glucose ester	344
483.1732	8.4	C_21_H_24_N_8_O_6_	343
447.1648	8.6	Kaempferol 7‐*O*‐glucoside	338
319.1264	3.8	5‐Pentadecylresorcinol	338
201.0444	1.0	Bergaptol	330
145.1007	9.9	Coumarin	326
205.1783	22.6	Acetyl eugenol	320
545.1999	11.1	Flavonol 3‐*O*‐[alpha‐l‐rhamnosyl‐(1‐>6)‐beta‐d‐glucoside]	318
389.1581	10.7	Laserolide	317
315.0974	3.9	Avenanthramide 2c	301
153.0332	4.4	Gallic aldehyde	291
123.0574	5.9	3‐Methylcatechol	287
391.1730	7.1	Viguiestenin	250

**Table A3:** Crude chemical composition of white birch bark methanol extract using UPLC‐QTOF‐MS analyses in positive mode

Exact mass [M+H]	Retention time (*R_t_*)	Compounds	Area under the curve
140.1349	11.6	Pinidine	5,190
128.1771	14.6	Coniine	3,912
150.9699	13.6	Asparagusate	3,106
377.3593	11.1	C_20_H_40_O_6_	3,809
326.9941	13.0	Butonate	2,951
122.8746	9.7	C_6_H_2_O_3_	2,613
342.2122	11.1	Isocorypalmine	3,314
242.4491	7.4	C_16_H_49_	3,462
216.4768	9.6	C_6_H_47_O_6_	3,991
215.0140	5.6	2‐Deoxy‐d‐ribose 1‐phosphate	1,947
276.1790	7.4	Annotinine	1,859
378.1298	11.2	6‐(2‐Hydroxyethyl)‐5,6‐dihydrosanguinarine	1,398
142.1624	10.8	Methylconiine	1,374
290.2941	9.3	17a‐Aza‐d‐homoandrost‐5‐en‐3beta‐ol	1,608
342.9818	11.1	Carbophenothion	1,201
300.0028	9.3	1‐Methylseleno‐*N*‐acetyl‐d‐galactosamine	1,181
285.1934	20.1	Retinal	1,112
352.8556	9.4	C_8_O_16_	935
388.1222	9.4	C_12_H_19_O_14_	823
243.1798	7.4	Falcarinone	878
217.1958	9.6	12‐Hydroxydodecanoic acid	541
251.3133	20.1	C_9_H_46_O_6_	939
217.1958	9.9	12‐Hydroxydodecanoic acid	523
227.9633	8.8	C_13_H_7_O_4_	709
159.0468	1.4	Ibotenic acid	482
276.9267	7.4	C_15_O_6_	228
285.1934	20.3	(+)‐Larreatricin	337
251.3133	20.5	C_10_H_34_O_6_	256
126.1111	3.3	C_4_H_13_O_4_	301

**Table A4:** Crude chemical composition of white birch bark methanol extract using UPLC‐QTOF‐MS analyses in negative mode

Exact mass [M−H]	Retention time (*R_t_*)	Compounds	Area under the curve
313.1478	11.6	Perilloside	1,893
271.0771	12.3	Naringenin, Phloretin, Butein ou Arbutin	1,594
295.1493	14.5	Deoxystansioside	1,284
373.1544	7.4	Hydroxymatairesinol	1,249
639.2776	11.1	C_42_H_40_O_6_	1,162
299.1744	14.2	Kaempferid	1,138
315.1700	10.8	Sesquiterpene	1,128
285.0872	15.7	Acacetine	1,128
329.2446	13.6	Dimethylquercetin	1,107
521.2055	9.7	C_33_H_30_O_6_	1,035
593.2709	11.1	Apigenin‐diglucoside	1,013
655.2672	8.9	Methyl 3‐*O*‐benzyl‐4‐*O*‐(2,3,4‐tri‐*O*‐benzyl‐b‐d‐xylopyranosyl)‐b‐d‐xylopyranoside	997
473.1739	9.5	Chicoric acid isomer	958
459.1974	7.3	Pentacosyl Resorcinol	950
573.1666	13.0	C_35_H_26_O_8_	936
287.0742	9.6	Eriodictylol isomer ou Phlorin	844
523.2272	9.4	Ligstroside isomer	804
523.2272	9.3	Ligstroside	785
431.1400	10.3	Apigenin 6‐glucoside	746
319.0613	7.1	3,4‐DHPEA‐EDA	693
421.1602	9.1	C_21_H_26_O_9_	670
423.1767	9.6	C_21_H_28_O_9_	657
473.1739	9.2	Chicoric acid	594
423.1767	9.9	C_21_H_28_O_9_	580
543.1417	12.3	Diferuloylquinic acid	567
435.1445	10.8	Phloridzin	492
343.1340	8.0	Anhydro‐secoisolariciresinol	464
327.1602	7.4	C_20_H_24_O_4_	450
421.1305	5.6	C_27_H_18_O_5_	444
627.3060	11.6	C_31_H_48_O_13_	437
331.1245	5.5	Galloyl glucose	412
295.1576	16.5	C_12_H_24_O_8_	397
507.2391	11.5	C_30_H_36_O_7_	395
469.1547	8.6	Valoneic acid dilactone	394
314.1710	11.6	C_16_H_27_O_6_	389
451.1421	9.6	Hydroxyphloretin‐glucoside	383
505.2050	7.3	C_26_H_34_O_10_	359
297.1543	15.3	C_15_H_22_O_6_	356
507.1955	10.0	C_32_H_28_O_6_	352
393.1194	13.9	C_22_H_18_O_7_	351
287.0824	11.0	Eriodictyol isomer or Phlorin	333
423.1866	10.3	C_22_H_32_O_8_	327
301.1008	13.3	Hesperetin	306
477.1890	10.2	Taramixin	304
179.0905	1.5	Caffeic acid	286
303.0838	8.5	Taxifolin	280
163.0530	7.9	Coumaric acid	268
377.1246	14.7	3,4‐DHPEA‐EA	239
296.1717	14.5	C_16_H_25_O_5_	236
137.0389	9.9	Hydroxybenzoic acid	228
377.1339	16.3	Oleuropein‐aglycone	223
137.0502	6.0	Hydroxybenzoic acid	195
327.1514	9.2	C_12_H_24_O_10_	133
393.1385	12.7	C_23_H_22_O_6_	118
521.2055	7.5	C_33_H_30_O_6_	105
